# Modification of Bulk Density, Flow Property and Crystallinity of Microcrystalline Cellulose Prepared from Waste Cotton

**DOI:** 10.3390/ma16165664

**Published:** 2023-08-17

**Authors:** Sabiha Tasnim, Md. Fazlul Karim Tipu, Md. Sohel Rana, Md. Abdur Rahim, Mithila Haque, Md. Shah Amran, Abu Asad Chowdhury, Jakir Ahmed Chowdhury

**Affiliations:** 1 Department of Pharmaceutical Chemistry, Faculty of Pharmacy, University of Dhaka, Dhaka 1000, Bangladesh; 2 Department of Pharmaceutical Technology, Faculty of Pharmacy, University of Dhaka, Dhaka 1000, Bangladesh

**Keywords:** microcrystalline cellulose, waste cotton, bulk density, flow property, crystallinity

## Abstract

The most affordable type of tablet is the immediately compressible tablet, which uses microcrystalline cellulose (MCC), a popular pharmaceutical excipient, as a filler or binder. To make it compatible with different active drugs and excipients, we tried to change some physical properties of the MCC. In the current study, we used a chelating agent to pretreat the waste cotton before pulping, bleaching, and finally, hydrochloric acid degradation with a concentration of 2N at 100 °C temperature for 20 min to prepare MCC. The prepared MCC was treated with different concentrations of sodium hydroxide at room temperature or at −20 °C followed by precipitation with hydrochloric acid or ethanol with complete washing with distilled water till neutralization. Evaluation of the degree of polymerization (DP) and FT-IR spectrum confirm the identity of the microcrystalline cellulose. The DP was found to be 216. The bulk density of the unmodified MCC was 0.21 while that of modified MCC varied from 0.253 to 0.594. The modified MCC powder showed good flow properties compared to the unmodified MCC as evaluated by the Hausner index, Carr’s index and the angle of repose. The scanning electron microscopy (SEM) of the MCC revealed that the rod shape has been changed to an oval shape due to treatment with sodium hydroxide at −20 °C. The X-ray crystallographic (XRD) analysis indicated that the unmodified MCC and standard MCC showed the crystallinity index (CrI) value of 86.82% and 87.63%, respectively, while the value ranges from 80.18% to 60.7% among the modified MCC powder. The differences in properties of the MCC might be due to the variation of rearrangement of the cellulose chain among the MCC particles due to treatment with different concentrations of a base at different temperatures and precipitation environments. This has enabled us to prepare MCC with different properties which might be compatible with different drugs.

## 1. Introduction

Cellulose is the most abundant polymer in the world and is composed of polymers of glucose linked with each other by β (1→4) glucosidic linkage. Microcrystalline cellulose (MCC) is partially a depolymerized cellulose isolated from pure α-cellulose. According to the United States Pharmacopoeia (USP) the degree of polymerization (DP) of MCC is less than 350 [1]. Commercially, it is prepared from hardwood [2], cotton [3] and different types of agro wastes such as rice straw [4], wheat straw [5], oil palp [6], jute [7], sugarcane baggage [8], corn cob [9], sisal fibers [10], etc. The cellulose from the above sources is purified by pulping methods and subjected to acid hydrolysis [11,12], extrusion [13], enzyme-mediated process [14] or steam explosion [15] for the preparation of MCC. Chemical treatment removes the amorphous region of the cellulose and thus leaving the MCC as a product. Because of its great compactness under low compression pressures, high binding capability and capacity to make tablets that are exceptionally hard, stable and disintegrate swiftly, MCC manufacturing has received a lot of interest for pharmaceutical usage [16,17]. MCC is rated as the most useful filler for the direct compression tableting process [18,19,20]. It also has the lowest friability, the highest intrinsic lubricity, and the greatest dilution potential of any binder. Despite a number of factors like low bulk density, poor flow property, high lubricant sensitivity, moisture content and compactness with active drugs limiting its use as a direct compressible excipient [21,22].

Direct compression is a preferred tablet manufacturing process and modern pharmaceutical companies are trying to increase their manufacturing output by reducing operating costs [23,24]. The bulk density, flowability and compressibility of the active drug-excipient mixture have a high impact on the choice of tableting process. The bulk density of a powder is a property that increases as the consolidated stresses go up. It influences the mixing property of one powder with another and variation may lead to segregation which ultimately causes variation in dose uniformity. Moreover, a powder mixture with low bulk density requires an excess force to apply for tablet formation while increased bulk density powder may be exposed to insufficient pressure, both may result in crushing or capping problems for tablets [25]. In contrast, the flow property is directly associated with the ease of transfer of powder mixture from the hopper to the die cavity in appropriate amounts and variation of this property affects the dose uniformity of tablet [26]. Additionally, crushing strength which depends upon the compressibility of the powder affects the disintegration and subsequent dissolution of the drug in the gastrointestinal tract. Hence, proper crushing strength is essential for quality products [25,26].

Traditional excipients have limited ability to provide desirable bulk density, flow property and crushing strength with cohesive active drug materials. Though widely used, MCC cannot be used with all drugs due to variations in particle size, bulk density and compressibility of the drug powder. No report has been published about the modification of the physical properties of MCC. In this study, we have proposed a new technique to modulate the bulk density, flow property and crystallinity of MCC by treating with different concentrations of sodium hydroxide solution at different temperatures followed by precipitation and oven drying.

## 2. Materials and Methods

### 2.1. Materials

The waste cotton utilized as raw materials in this investigation was purchased from Badsha Textiles Limited, Bhaluka, Mymensingh, Bangladesh. Standard MCC (Avicel^®^ PH 102) having an average particle size of 100 μm, was collected from the local supplier of Sigma Aldrich, St. Louis, MO, USA. CUEN solution (Cupriethylenediamine solution) was purchased from a local supplier of Fisher Scientific, Waltham, MA, USA. The rest of the reagents used for synthesis in the laboratory were of reagent grade.

### 2.2. Preparation and Modification of Prepared MCC

A sample of 30 g of waste cotton was steeped for 1 h at 100 °C in a medium containing sodium hydroxide (1%), sodium dodecyl sulfate (0.33%), and EDTA (0.085%). The process was repeated once. The treated sample was then soaked in a 2.5% solution of sodium hypochlorite at 60–70 °C for 1 h. After bleaching and vigorous washing for complete neutralization, it was again treated with sodium hydroxide (17.5%) solution at 100 °C for 1 h. After washing with distilled water till neutralization, the isolated alpha cellulose was exposed to acid hydrolysis with 2N HCl for 20 min. The prepared microcrystalline cellulose was filtered and washed with distilled water till neutralization. The collected powder was dried at 60° to 70 °C overnight, passed through a 125-micron sieve, assigned as unmodified MCC (UM-MCC) and stored at room temperature (RT) until use.

To change the physical nature, the prepared microcrystalline cellulose (MCC-UM) was basified with 20% NaOH solution to make a slurry and kept at RT for 4 h (cellulose weight to volume of sodium hydroxide ratio was 1:6). The slurry was then neutralized with 1N hydrochloric acid (MCC-M1) or diluted by addition of ethanol (MCC-M2) where ethanol should be 50% of total final volume. The MCC-UM sample was also subjected to soaking in 20% sodium hydroxide at −20 °C for 4 h with subsequent treatment with 1 N HCl (MCC-M3) or ethanol (MCC-M4) as above. The other part of MCC-UM was also treated with 8.5% NaOH to make a suspension and kept at −20 °C for 4 h followed by adding ethanol as above at RT (MCC-M5). The MCC slurry after HCl or ethanol treatment was filtered and washed several times with distilled water to make it neutral. The powder was then dried at 60–70 °C overnight. The dried mass of cellulose was crushed to make a powder. The MCC powder which passed through a 125 mesh sieve but was retained on a 200 mesh sieve was collected.

### 2.3. Identification of Microcrystalline Cellulose by Chemical Test

For chemical identification, each prepared and modified MCC sample (10 mg) was disseminated in two milliliters of iodinated zinc chloride solution comprising zinc chloride (1.9 g/mL), potassium iodide (0.62 g/mL) and iodine (0.05 g/mL) [1]. The presence of starch was also evaluated by using an iodine solution to treat MCC powder.

### 2.4. Identification of MCC by Determining Degree of Polymerization

Normally, the degree of polymerization (DP) of the MCC sample is simply measured from the value of intrinsic viscosity solution in 0.5M copper (II) ethylenediamine solution. Different amounts were dissolved to prepare various concentrations of prepared unmodified MCC (0%, 0.0625%, 0.125%, 0.25%, 0.5%, 1.0%, and 2.0%). At 25 °C, the time required to move the MCC solution surface between the Ostwald Viscometer 1831 levels was measured. A densitometer was also used to determine the density of the MCC solution. The relative viscosity of MCC was calculated followed by calculating the reduced viscosity. The value of intrinsic viscosity of the MCC was obtained from the y-intercept value of the plot of reduced viscosity vs. concentration of MCC solution. The degree of polymerization was determined using the following equation based on the intrinsic viscosity value [27].

(DP)^0.85^ = 1.1 × η (where η is the intrinsic viscosity)

### 2.5. Identification of MCC by FT-IR

The MCC sample was blended with KBr (0.1% MCC) and finely ground before being crushed to form a disc. In transmission mode, an FTIR 8400S SHIMADZU spectrophotometer (Kyoto, Japan) was used to record FT-IR spectra in the frequency of 4000–400 cm^−1^ with a resolution of 1 cm^−1^. A total of 30 scans were taken in total.

### 2.6. Evaluation of Bulk Density and Tapped Density of the Unmodified and Modified MCC

A specific amount (10 gm) of the unmodified and modified MCC was drained into a measuring cylinder (50 mL) to determine the bulk volume followed by calculating the bulk density from the ratio of weight to bulk volume of each sample. After 200 taps of the cylinder by the instrument (Digital Automatic Tap/Bulk Density Test Apparatus, Model: VTAP-MATIC, Veego, Karnataka, India), the tapped volumes, V_200_, were determined for each sample. The tapped density of each sample was calculated by dividing the weight (10 gm) by the tapped volume [28]. Each experiment was repeated three times and calculated the average value with standard deviation.

### 2.7. Analysis of Flow Property by Measuring Hausner Index and Compressibility Index (Carr’s Index)

Using the same equations as described before [29], Carr’s index and the Hausner index were calculated from the value of bulk and tapped density.

### 2.8. Analysis of Flow Property by Measuring the Angle of Repose

The angle of repose of the MCC powder was determined by transferring the powder through a funnel clamped above a horizontal surface for 3 cm. A graph sheet was placed under a funnel that was clamped above a horizontal surface for 3 cm to determine the angle of repose of the MCC samples. The funnel’s tube was used to transfer the MCC powder. The angle of repose of the MCC powder was calculated using the equation tanθ = 2 h/D where h stands for the height of the pile of MCC powder, D for the diameter of the base of the pile of powder and θ for the angle of repose [30]. Each experiment was repeated thrice and the average angle of repose value was calculated with standard deviation.

### 2.9. Morphology Analysis by Scanning Electron Microscopy (SEM)

SEM photograph of the prepared and modified MCC was taken with the help of JEOL JSM-6490LA (Akishima, Japan), an analytical scanning electron microscope at 500-fold magnification. The samples were seated upon the specimen surface with double-sided adhesive tape followed by the application of electrically conductive coating [31].

### 2.10. Evaluation of Crystallinity of the MCC by X-ray Diffraction

The crystal structure of MCC samples (150 mg) was investigated using an X-ray diffractometer (D/MAX-1200, Rigaku, Tokyo, Japan), with diffraction angles 2θ ranging from 6 to 40 degrees. To measure the crystallinity of materials, a profile analysis was performed using a peak fitting tool using Gaussian line forms. With the help of a 10 kV X-ray generator (Rigaku RINT2000), the X-ray diffraction pattern was obtained using Kb-filtered Cu kα radiation. Using the following equation, the height of the 200 peak (I_200_, 2θ = 22.5°) and the minimum elevation between the 200 and 100 peaks (I_am_, 2θ = 18°) were used to calculate the Crystallinity index (CrI) of the MCC [32].

CrI (%) = 100 * (I_200_-I_am_)/(I_200_)

## 3. Results and Discussion

### 3.1. Preparation and Chemical Identification of the MCC

The MCC nature of the prepared sample was confirmed by the presence of a violet—blue coloration when it was subjected to iodinated zinc chloride solution while the absence of starch in the sample was confirmed by the non-appearance of any brown coloration when the sample was treated with the iodine solution.

### 3.2. Identification of the MCC by Degree of Polymerization of the MCC

**Figure 1:**
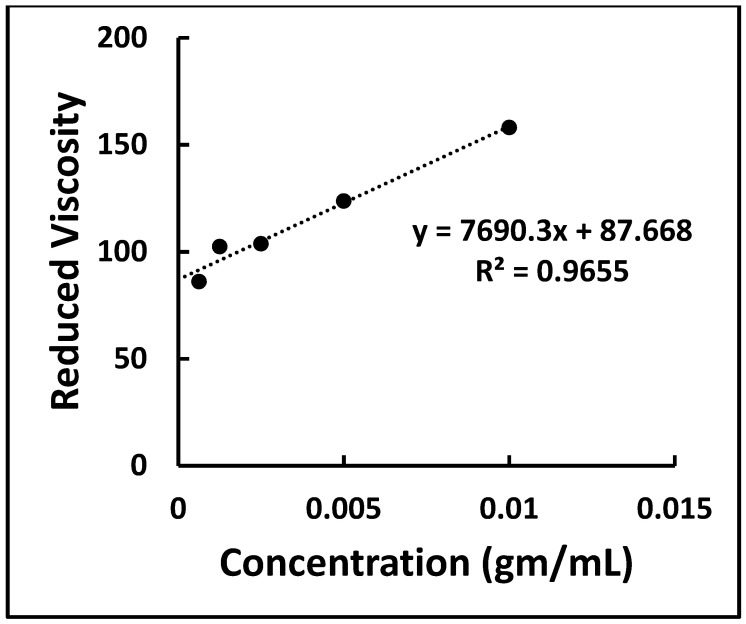
Determination of degree of polymerization (DP) of prepared MCC.

The time to move the prepared MCC solution with different concentrations between the surface levels of the Ostwald Viscometer 1831 was determined followed by the calculation of the corresponding reduced viscosity. The intrinsic viscosity was found to be 87.668 at the y-intercept of the graph of reduced viscosity vs. concentration (Figure 1). The degree of polymerization of prepared MCC was calculated to be 216. According to USP, MCC should have a DP of less than 350 [1]. This ensures that the prepared sample is MCC once more.

### 3.3. Identification of the MCC by FT-IR of MCC

**Figure 2:**
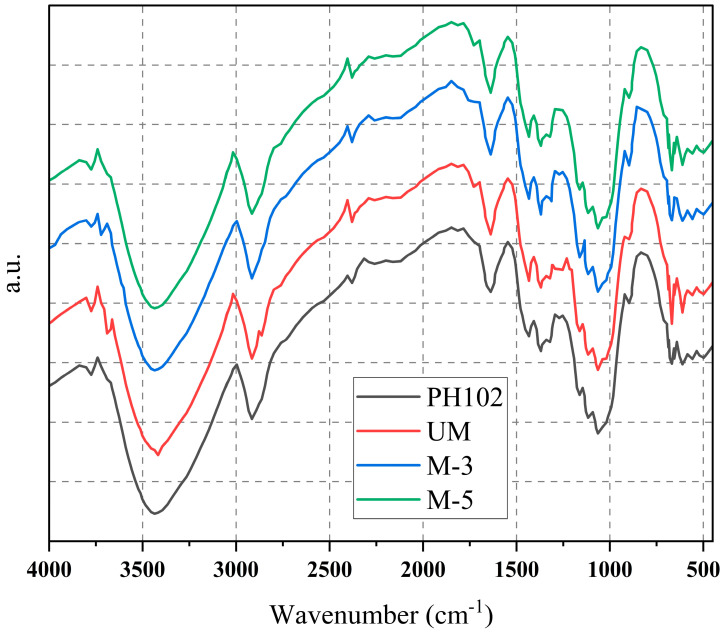
FT-IR spectra of Avicel PH102, unmodified MCC (UM), modified MCC-M3 (M-3) modified MCC-M5 (M-5).

In the FT-IR spectra of Avicel PH102, the O-H stretching absorption varied from 3423.25 to 3433.29 cm^−1^, the C-H stretching absorption ranged from 2914.44 to 2918.30 cm^−1^, and the C-O-C stretching absorption ranged from 1058 to 1112 cm^−1^. These absorptions are comparable to cellulose backbone absorptions in general [33,34]. The presence of all the peaks for the modified and unmodified samples are same as that of the standard Avicel PH102. This clearly indicates that all are the same and modification did not interfere with the basic chemical structure of the MCC. The appearance of the absorption peak at 1740 cm^−1^ in the spectrum of standard and MCC-UM indicates the presence of hemicellulose in the microcrystalline cellulose (Figure 2). In contrast, the absence of the same peak in the FT-IR spectrum of MCC-M3 and MCC-M5 suggests that these samples do not contain any hemicellulose [34]. This might be due to the solubilization of hemicellulose by sodium hydroxide. Furthermore, the peak at 1595 cm^−1^ is linked to an aromatic ring stretch [35]. which is strongly linked to the aromatic C–O stretching mode and indicates the presence of lignin. The absence of this peak in the FT-IR spectrum confirms the absence of lignin in the MCC sample. This also confirms that the prepared MCC is extremely pure. The 3600–3100 cm^−1^ region of the OH-stretching vibration broad band contains a lot of information about hydrogen bonding. The peak indicative of hydrogen bonding in the standard and MCC-UM spectra increased larger and more intense indicating a high crystallinity zone.

Additionally, the peak migrated to higher wave number values in amorphous samples [35]. The modified MCC shows shifting of the peak toward a higher wave number (3433 for MCC-M3 and 3429 cm^−1^ for MCC-M5) compared to that of standard and unmodified MCC (3425 and 3423 cm^−1^) indicating a lower crystallinity of the modified MCC which might be due to the breakdown of the crystalline region by treatment with sodium hydroxide. 

### 3.4. Evaluation of Flow Property (CI, Hausner Index, Angle of Repose) of the Unmodified and Modified MCC

Bulk density is the degree of how fine a powder can be filled in a constrained space on recurrent tapping, whereas tap density is the extent to how well a powder can be filled in a restricted space from a hopper into the die chamber of a rotary tablet compression machine. In general, the higher a material’s bulk and tapped densities are, the better its ability to flow and rearrange under compression. The bulk density of the prepared MCC (MCC-UM) is 0.210 which is much lower than that of standard Avicel PH102 (Table 1). Treatment of the MCC-UM with 20% sodium hydroxide at RT followed by precipitation of the MCC by 1 N acid (MCC-M1) or ethanol (MCC-M2) treatment increases the bulk density to 0.303 and 0.253, respectively.

Again, treatment of the MCC-UM with 20% sodium hydroxide at −20 °C followed by precipitation by 1 N acid (MCC-M3) or ethanol (MCC-M4) treatment enhances highly the bulk density to 0.593 and 0.473, respectively. This indicates that the lower the treatment temperature, the higher the bulk will be as well as the tapped density of the modified MCC. It has been reported that the intermolecular hydrogen bonds of cellulose at low temperatures in the presence of sodium hydroxide are weakened or broken which increases the possibility of interaction between the cellulose and water molecules [36,37,38]. As a result, the cellulose chain might undergo dissolution in water depending on the molecular weight, temperature and concentration of sodium hydroxide. In our cases, treatment of MCC with 20% sodium hydroxide at −20 °C makes the slurry very viscous, not a clear solution, which might be due to the weakening of the intermolecular hydrogen bonds of the MCC. The subsequent processing with the hydrochloric acid to neutralize the medium or with ethanol followed by water wash for neutralization might rearrange the cellulose chain in such a way as to change the shape that enhances the bulk density. In addition, treatment of the MCC-UN with 8.5% sodium hydroxide at −20 °C followed by precipitation by ethanol (MCC-M4) treatment also enhances the bulk density (0.433) and tapped density (0.553) though the value is lower compared to that of 20% sodium hydroxide treatment. A lower concentration of sodium hydroxide in this case might weaken the intermolecular hydrogen bond of the cellulose chain in MCC to a lower extent that might change the bulk and tapped density to a lower extent [35,36].

Flow property is an important parameter for pharmaceutical excipients. The powder should have the desired flow property to be used as a pharmaceutical excipient. The Hausner index (HI) is related to inter-particle friction and this can be used to predict the powder flow. The values of HI of standard Avicel PH102, prepared unmodified MCC (MCC-UM), modified MCC-M1, MCC-M2, MCC-M3, MCC-M4 and MCC-M5 were 1.368 ± 0.056, 1.428 ± 0.041, 1.374 ± 0.047, 1.355 ± 0.008, 1.167 ± 0.004, 1.232 ± 0.017 and 1.277 ± 0.010, respectively (Table 1). The HI of prepared MCC (MCC-UM) is higher compared to all the modified MCC while the HI value of standard Avicel PH102 is very similar to that of the modified MCC-M1 and MCC-M2. In contrast, MCC-M3, MCC-M4 and MCC-M5 showed a lower value of HI compared to all the samples suggesting good flow properties of the modified MCC compared to all others. On the other hand, Carr’s percent compressibility index (CI) is also an indirect measurement of powder flow ability [39]. The powder compressibility index, calculated from measured bulk density and tapped density, of the standard, unmodified and modified MCC has been shown in Table 1. The values of CI of standard Avicel PH102, prepared unmodified MCC (MCC-UM), modified MCC-M1, MCC-M2, MCC-M3, MCC-M4 and MCC-M5 were 26.801 ± 3.087, 29.957 ± 2.056, 27.188 ± 2.517, 26.218 ± 0.437, 14.428 ± 0.316, 18.834 ± 1.084 and 21.698 ± 0.594, respectively. A CI value of less than 20 is an indication of the good flow ability of powder [28]. This indicates that the flow property of MCC-M3 and MCC-M4 is fairly good.

**Figure 3:**
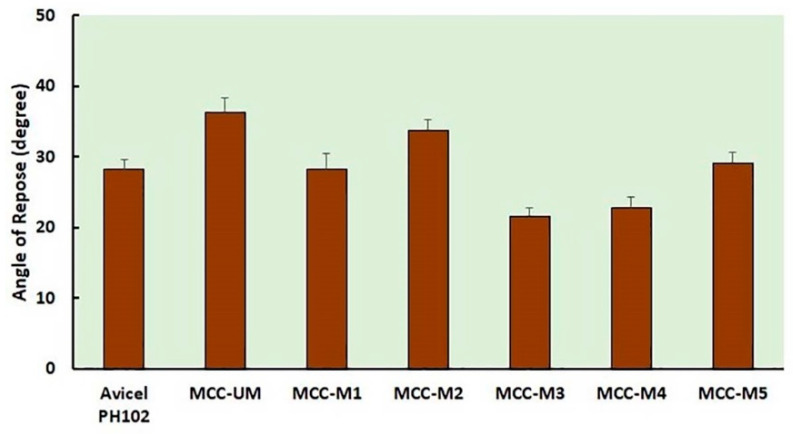
Angle of repose of the standard, unmodified and modified MCC.

The angle of repose (AoR) is an indirect method of qualifying powder flow ability because of its relationship with inter-particle cohesion. As a general rule, powders with an angle of repose lower than 25° show excellent flow property while 25–30° shows good flow property. The angle of repose higher than 40° indicates very poor flow ability [40]. The angle of repose of prepared MCC is lower than 25° indicating an excellent flow property of the powder. The unmodified MCC showed the highest value of AoR indicating comparatively poor flow property. MCC-M3 and MCC-M4 show good flow properties while other samples show passable flow properties based on the value of the AoR (Figure 3). This result agrees with the previous result of the flow property of all unmodified and modified MCC based on CI and the Hausner index. The variation in flow properties of the modified and unmodified MCC sample might be occurred due to the variation in particle size, shape and surface texture [41,42].

### 3.5. Evaluation of MCC by SEM

**Figure 4:**
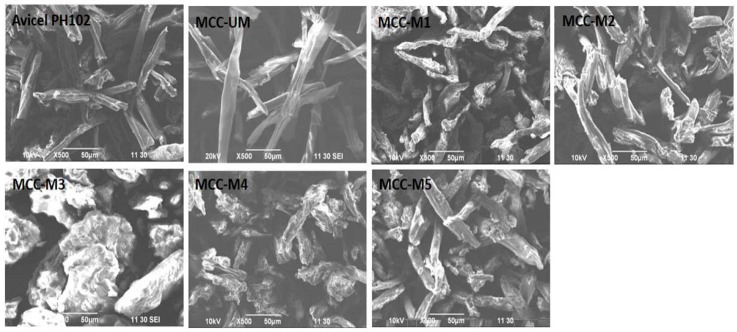
Scanning electron micrograph of Avicel PH102, unmodified and modified MCC.

The SEM photomicrograph showed that the particles of unmodified MCC are different from all the modified ones. The unmodified MCC is rod shaped. The MCC-M1, MCC-M2 and MCC-M5 are rod to oval in shape while the MCC-M3 and MCC-M4 are oval to spherical in shape (Figure 4). All the samples showed almost the same particle size though different in shape. The treatment of the unmodified MCC with 20% sodium hydroxide at different temperatures might hydrolyze and dissolve the hemicellulose resulting in the variation of surface texture as shown in Figure 4.

Moreover, sodium hydroxide (20%) treatment at low temperature (−20 °C) might weaken the interchain hydrogen bonding of MCC making the viscous slurry. Subsequent treatment with HCl or ethanol followed by washing till neutralization might rearrange the cellulose chain in such a way as to modify the shape of the MCC [36,38]. The rearrangement of the cellulose chain here might be responsible for the spherical shape of the MCC-M3 and MCC-M4. In contrast, other samples were treated in 8.5% sodium hydroxide medium or at room temperature. This might not be able to rearrange the cellulose chain to a high extent. The low extent rearrangement might be responsible for the lower extent of shape change in MCC-M1, MCC-M2 and MCC-M5. The spherical shape of the MCC-M3 and MCC-M4 might be responsible for the better flow property which was confirmed by Carr’s index, Hausner Index (Table 1) and AoR (Figure 3) value. On the other hand, the unmodified MCC (MCC-UM) due to its rod shape structure might be responsible for the poor flow property.

### 3.6. Evaluation of Crystallinity of the MCC by X-ray Diffraction

**Figure 5:**
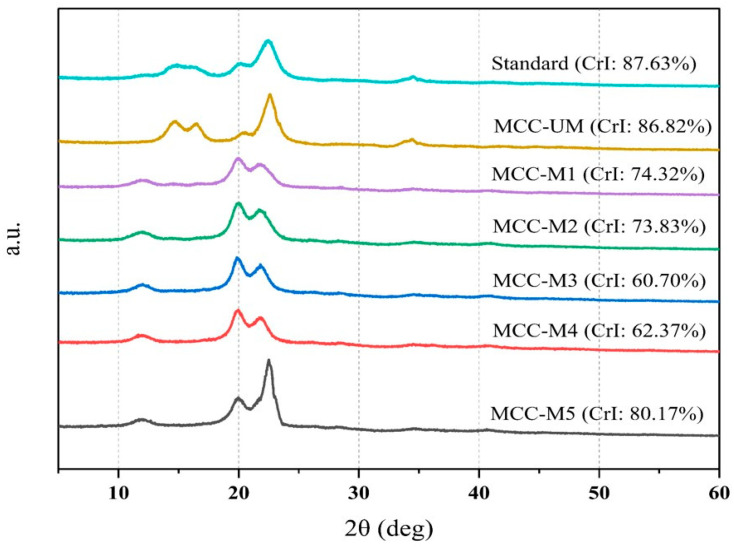
Characterization of the standard, unmodified and modified MCC by XRD analysis.

The crystalline features of cellulose are influenced by its mechanical and thermal properties. The reinforcing ability and mechanical strength of cellulose, in particular, are deciding factors in its application in pharmaceutical technologies. The cellulose XRD diffraction patterns were obtained at 2θ = 22.5° which correspond to the lattice planes 200 in the cellulose lattice (Figure 5). The presence of crystalline cellulose [43,44] was confirmed by the occurrence of a large crystalline peak at 22.5° in all the samples including standard, unmodified and modified MCC. The percent crystallinity indexes (CI) of the standard MCC (Avicel PH101), MCC-UM, MCC-M1, MCC-M2, MCC-M3, MCC-M4 and MCC-M5 were 87.63, 86.82, 74.23, 73.83, 60.70, 62.37 and 80.18, respectively. Comparing the CI%, it can be inferred that treatment with sodium hydroxide at a high concentration decreases the crystallinity of all MCC to a high extent. Moreover, treatment of MCC at low temperatures with the same concentration of base reduces the crystallinity of MCC to a high extent as found in the %CrI value of MCC-M3 and MCC-M4 if compared with that of MCC-M1 and MCC-M2. In contrast, the difference in %CI value between sample MCC-M1 and MCC-M2 is negligible and the same is found when comparing the %CI value between MCC-M3 and MCC-M4 indicating that the recrystallization agents (hydrochloric acid and ethanol) do not have any impact on the crystallinity of the modified MCC.

A prominent crystalline peak at 2θ = 19.8° in sample MCC-M1, MCC-M2, MCC-M3 and MCC-M4 is evident (Figure 5). This indicates the transformation of cellulose I to cellulose II in all the above samples which might the due to the treatment of MCC with sodium hydroxide. Base treatment of MCC with high NaOH concentration at RT or low temperature causes sodium hydroxide solution penetration to the amorphous regions located among the crystal regions of MCC. The permeation of the basic solution in the less orderly areas induces the formation of Na-cellulose II. However, the development of Na-cellulose II with anti-parallel chains is thermodynamically advantageous, as it results in the steady reduction of cellulose I crystalline areas, which leads to the formation of Na-cellulose II crystallites [44,45]. After diluting with ethanol or neutralizing the base with hydrochloric acid followed by washing with distilled water, the base is completely removed and the cellulose II type MCC might remain. This study revealed that this low crystallinity in the modified MCC might be responsible for the high disintegration power of modified MCC. In contrast, low concentration (8.5%) sodium hydroxide treatment does not cause the transformational change from cellulose I to cellulose II to a high extent as evident in MCC-M5.

## 4. Conclusions

The present work was undertaken with the motive of utilizing waste cotton materials to prepare microcrystalline cellulose with modification of bulk density, flow property, shape and crystallinity. Treatment of the prepared MCC with different concentrations of sodium hydroxide at different temperatures followed by mixing the MCC slurry with water, hydrochloric acid or ethanol enabled enhancement of the bulk density up to 0.594. The flow property of the modified MCC powder was high compared to that of unmodified MCC as evidenced by the value of the Hausner index, Carr’s index and angle of repose. The SEM photomicrograph showed that the shape of the particles of modified MCC ranges from rod to spherical shape which is responsible for the enhanced flow property of the modified MCC. We were also able to modify the crystallinity index of MCC ranging from 62.70% to 80.17% by treating the prepared MCC with a base at different temperatures. Taken together, it can be concluded that the MCC with different bulk densities with good flow properties and crystalline indexes can be prepared using the new method.

## Figures and Tables

**Table 1:** Bulk density, tapped density, Hausner ratio and compressibility index of the standard MCC, unmodified and modified MCC.

Sample	Bulk Density	Tapped Density	Hausner Ratio	Compressibility Index
Standard (Avicel PH102)	0.327 ± 0.006	0.447 ± 0.006	1.368 ± 0.056	26.801 ± 3.087
MCC-UM	0.210 ± 0.010	0.300 ± 0.017	1.428 ± 0.041	29.957 ± 2.056
MCC-M1	0.303 ± 0.012	0.417 ± 0.012	1.374 ± 0.047	27.188 ± 2.517
MCC-M2	0.253 ± 0.006	0.343 ± 0.006	1.355 ± 0.008	26.218 ± 0.437
MCC-M3	0.593 ± 0.015	0.693 ± 0.015	1.167 ± 0.004	14.428 ± 0.316
MCC-M4	0.473 ± 0.012	0.583 ± 0.021	1.232 ± 0.017	18.834 ± 1.084
MCC-M5	0.433 ± 0.015	0.553 ± 0.015	1.277 ± 0.010	21.698 ± 0.594

## Data Availability

Raw data of this article are available upon request from the authors.
